# Polydatin for treating spinal cord injury: Multiple mechanisms and challenges

**DOI:** 10.1016/j.jpha.2025.101451

**Published:** 2025-09-15

**Authors:** Zhishuo Wang, Jiaming Zhang, Longyu Li, Yuhao Zhang, Haoyu Shen, Chunfeng Shang, Zikuan Leng, Guowei Shang, Hongwei Kou, Keya Mao, Hao Han, Songfeng Chen, Hongjian Liu

**Affiliations:** a Department of Orthopedics, The First Affiliated Hospital of Zhengzhou University, Zhengzhou, 450052, China; b Department of Orthopedics, Beijing Tsinghua Changgung Hospital, School of Clinical Medicine, Tsinghua University, Beijing, 102218, China; c Department of Pediatrics, The First Affiliated Hospital of Zhengzhou University, Zhengzhou, 450052, China; d Department of Orthopedics, The Chinese PLA General Hospital, Beijing, 100039, China

**Keywords:** Polydatin, Spinal cord injury, Anti-inflammatory, Antioxidative stress, Neuroprotection

## Abstract

Spinal cord injury (SCI) is a serious neurological system disease. After SCI, a series of cascade reactions can cause irreversible damage, with a high disability rate and mortality rate. The complexity of the pathological mechanism of SCI limits the efficacy of various traditional therapies, and it is urgent to find new therapeutic means. In recent years, the method of extracting effective components from natural Chinese herbs for treating diseases has attracted widespread attention. Polydatin (PD) is an active ingredient extracted from Polygonum cuspidatum and its structure is similar to the traditional drug resveratrol. Sufficient studies have proved that PD plays anti-inflammatory, antioxidant, anti-apoptotic and neuroprotective roles in the treatment of multisystem diseases. These effects are also significant in the treatment of SCI. Based on the structural differences between PD and resveratrol, this paper illustrates the feasibility of PD in the treatment of SCI, and systematically expounds the pathophysiological process of SCI and the molecular mechanism of PD in the treatment of SCI. Furthermore, this article discusses feasible measures to improve the bioavailability of PD, summarizes the application of new drug delivery systems in PD, and analyzes the challenges and prospects of PD in SCI treatment.

## Highlights

• Polydatin has been widely used in various systemic diseases by virtue of its anti-inflammatory, antioxidant and anti-apoptotic properties.
• This paper reviews the experimental study of polydatin in the treatment of spinal cord injury.
• The challenges and feasible strategies of polydatin in the treatment of spinal cord injury are discussed.

## Graphical abstract

**Figure:**
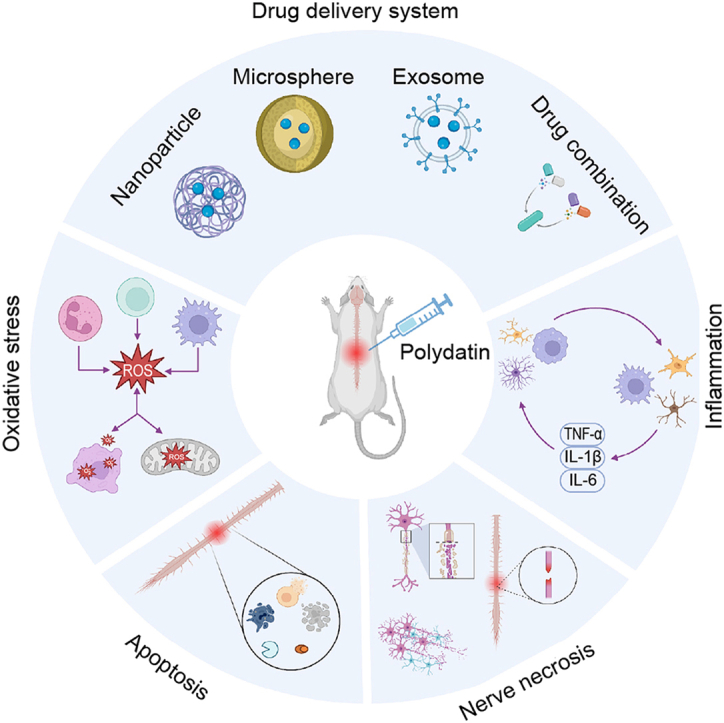

## 1 Introduction

Spinal cord injury (SCI) is a central nervous system (CNS) injury with serious consequences that can be fatal in severe cases. China and United States are both countries with a high incidence of SCI. In the two decades from 2000 to 2021, the total incidence of SCI in China reached 23.77 cases per million people, of which the incidence of traumatic SCI has remained at a high level of between 20 and 45 per million people [1]. Whether it's treatment or subsequent rehabilitation, it will impose a heavy social and economic burden on patients and families because of the permanence of SCI. According to statistics, in the United States, each patient spends between 1.1 and 4.7 million dollars on treatment for SCI in their lifetime [2].

SCI not only brings such a huge cost to society but also poses challenges to the medical community. A large number of patients face problems related to motor and sensory impairments because of the limited intrinsic repair ability of the CNS. Although there are therapeutic measures such as molecular analysis, drug interventions, stem cell therapy, and surgery with the development of the scientific society, these have many limitations, such as efficacy and controllability of the treatment [3]. Although methylprednisolone is commonly used in clinical treatment of SCI, it not only brings economic burden, but also leads to gastrointestinal bleeding, respiratory infection, sepsis, pulmonary embolism and other adverse reactions [4].

Based on the current situation, the search for new treatments and the use of natural herbal extracts deserves special attention. Polydatin (PD), also named piceid (3,4’,5-trihydroxystilbene-3-β-D-glucoside), is a compound isolated from Polygonum cuspidatum Sieb. et Zucc. Grapes, peanuts, red wine, hop granules, cocoa-containing products, chocolate products, and many diets are also sources of PD [5]. Modern studies have confirmed that PD can achieve antioxidant, anti-inflammatory, anti-ischemic injury, neuroprotection, anti-tumor, liver protection and other effects. Among them, antioxidant, anti-inflammatory and neuroprotection are essential in the treatment of SCI, and many researchers have conducted relevant experimental studies to prove the effectiveness and mechanism of PD in the treatment of SCI.

This paper introduces the pharmacokinetic characteristics and drug delivery methods of PD, systematically expounds the pathophysiological process of SCI, as well as the mechanism and research progress of PD in the treatment of SCI. In addition, in view of how to improve PD bioavailability, we summarize a series of methods through new material loading and drug combination, which has certain value for PD future research.

## 2 Characteristics of PD

### 2.1 PD and resveratrol

Polygonum cuspidatum Sieb. et Zucc.is a perennial herb, mainly distributed in China, Korea, and Japan. In China, it is primarily found in regions such as Shaanxi and Gansu. The dried roots of Polygonum cuspidatum have been widely used in traditional Chinese medicine for treating conditions such as suppurative dermatitis, favus, athlete's foot, gonorrhea, and hyperlipidemia, as recorded in the Compendium of Materia Medica [6]. Polygonum cuspidatum contains various biologically active compounds, among which resveratrol and PD are the two most important active compounds. PD, also named piceid, has a molecular weight of 390.4 g/mol. It is a natural precursor and glycoside form of resveratrol, possessing a monocrystalline structure. Both PD and resveratrol exist in cis and trans forms, with the trans isomers exhibiting greater biological activity than the cis isomers. Trans-PD is characterized by a trans-resveratrol molecule substituted at the 3-position with a β-D-glucosyl residue. It is classified as a stilbenoid, a polyphenol, a β-D-glucoside, and a monosaccharide derivative [7]. The difference in structure makes the pharmacological effects of the two both similar and unique, which can produce different effects in the treatment of diseases. For example, while resveratrol is primarily known for its anti-cancer properties, PD may be more effective at reducing inflammation, providing antioxidant effects, and offering neuroprotection. Modern studies have confirmed that PD exhibits a variety of pharmacological effects, including liver protection, cardiovascular system protection, lung protection, anti-tumor activity, anti-inflammatory effects, antioxidant effects, and antibacterial activity [8,9]. In an experiment comparing the antioxidant effects of PD and resveratrol on doxorubicin-induced oxidative stress cardiomyopathy in mice, PD demonstrated superior antioxidant activity, particularly in the removal of superoxide anions (O_2_^−^) and hydroxyl radicals (·OH) [10]. Glycosylated resveratrol brings higher antioxidant properties and photochemical stability, which gives PD greater potential in the treatment of SCI. In addition, PD has several unique advantages. PD is hydrolyzed to resveratrol in the gut by β-glucosidase of the intestinal flora, which prolongates the time of drug action and achieves targeted release. The targeted release ability of PD makes it play a more prominent role in mitochondrial protection and blood-spinal barrier repair [11]. The glycosylation structure of PD significantly improves the photostability and chemical stability of PD, which makes PD more suitable for long-term storage and formulation development. In addition, PD is more water-soluble than resveratrol, which enhances its suitability for use in injectable or oral solutions. Compared with resveratrol, the lower fat solubility promotes the absorption of PD through the lymphatic system and reduces the first-pass effect of PD in the liver. In general, PD has high bioavailability and chemical stability, slow metabolic efficiency, and a long half-life, which helps to maintain a stable blood concentration. Compared with resveratrol, PD may be worth further investigation.

### 2.2 Some methods to increase PD production

To explore the distribution of PD content in Polygonum cuspidatum, researchers used sequencing technology to obtain the genomic map of Polygonum cuspidatum, which showed that the expression levels of 2173 genes in the roots of Polygonum cuspidatum were higher than those in the ground tissues, indicating that the roots of Polygonum cuspidatum are the main medicinal tissues. Common methods of producing PD include plant extraction and chemical conversion. From a botanical point of view, certain treatments of crops containing PD can help to increase PD content. For instance, in a study on plant protectants, vines treated with Tessior at the dormant bud or visible inflorescence stages showed significantly higher PD concentrations compared to untreated vines [12]. Additionally, research on Polygonum cuspidatum seedlings revealed that under drought stress conditions, exogenous application of 100 mM melatonin, an indole heterocyclic compound, acts as a biostimulant to enhance growth, leaf gas exchange, antioxidant enzyme activity, and PD content [13]. Nitrogen content in crop fertilization also influences the levels of various plant components. It was found that reducing nitrogen application significantly decreased the fresh weight and yield of dormant shoots. However, nitrogen reduction treatment notably increased the content of PD and other phenols in grapes, regardless of the season. This highlights the importance of reducing nitrogen application during the grape growing season to enhance PD content [14]. Furthermore, an appropriate concentration of phosphorus (P) is beneficial for crop growth and the accumulation of active components. A 0.2 M P level supply combined with inoculation of Bryococcus was shown to promote PD concentration in knotweed, providing a reference for increasing PD levels through trace element supplementation and microbial inoculation [15]. In addition to plant-based methods, microbes also have the potential to synthesize PD. Due to the PD's crab-negative properties and the high availability of malonyl coenzyme A, Yersinia lipolytica has been selected for PD production. Shang et al. [16] achieved up to 6.88 g/L PD production in Yarrowia lipolytica by optimizing glucose concentration and introducing two nutrient marker genes.

Glycosylation is one of the most effective biocompatible methods to improve water solubility, bioactivity and structural diversity of natural products, and has received great attention in biosynthesis and chemical transformation [17]. Similarly, researchers have conducted many studies on the generation of PD by glycosylation of resveratrol. The traditional synthesis method is to glycosylate resveratrol with uridine diphosphate dependent glycosyltransferase (UGT (BS)), but it is difficult to accurately glycosylate on C3 hydroxyl group and produce PD. Zhu et al. [18] successfully applied molecular evolution to UGT (BS) and developed a triple mutant (Y14I/I62G/M315W) that achieves over 90% 3-OH glycosylation of resveratrol, with PD as the predominant product. Based on transcriptome analysis of Polygonum polygonum, Liu et al. [19] identified the enzyme Pcus_1822 with glycosyl catalytic activity, and through engineering design and fermentation optimization, the yield of PD reached 545 mg/L.The above indicates that there are many ways to increase the yield of PD by plant extraction or chemical synthesis, which is a promising direction for research.

### 2.3 Pharmacokinetics of PD

Before drugs are used in basic research and clinical research, it is particularly crucial to understand their pharmacokinetics. The absorption, distribution and metabolism of PD are closely related to its biological activity. Studies have shown that resveratrol penetrates cell membranes through passive transport, and after PD enters the human body, it can be absorbed through two different transport systems: passive diffusion and active transport. The latter mainly relies on sodium-dependent glucose transporters (SGLT) and mainly exists in the gastrointestinal tract [20]. The rate of PD absorption is temperature-dependent: the absorption rate at 4 °C is 1.6 times lower than that at 37 °C, which confirms its dependence on the active transport of SGLT1 [21]. PD has a significant transepithelial transport effect and is highly absorbed by the human body and the permeability coefficient is approximately 10 × 10^−6^ cm/s of root apical to basalateral flux [22]. To gain a more accurate understanding of the metabolism and distribution of PD in animals after administration, researchers determined the content of polydanosine in rat plasma and tissues by high performance liquid chromatography. After the researchers orally administered 50 mg/kg PD to the rats and intravenously injected 20 mg/kg PD, the concentrations of PD in the heart, liver, lung and kidney tissues of the two groups of rats were measured multiple times respectively to determine the metabolic distribution trend of PD. The result trends of both groups indicated that the concentration of PD reached the maximum value in each tissue 10 min after administration, and there were significant distribution differences among the tissues. Taking intravenous injection as an example, the highest content in liver and kidney tissues after administration was 5.22 ± 0.46 and 6.41 ± 0.77 μg/g, which was dozens of times that in cardiac tissues. This indicates that PD is mainly metabolized through the liver and kidneys. Sixty minutes after administration, the concentration of PD drug in the intestine reached the maximum value (6.69 ± 0.41 μg/g), which meant that there was enterohepatic circulation after intravenous administration [23].

Regarding the excretion of PD, the concentration of PD in each tissue decreased to the initial concentration 120 min after administration and was completely eliminated from the body 4 h after administration. In the study of rat excretion, the cumulative excretion rate of PD in urine within 96 h was 1.51%, which was more than 40 times that in feces. This indicates that PD is mainly excreted through urine [24]. After oral administration of PD, resveratrol was detected in the urine of rats, indicating that PD can be metabolized into resveratrol and exert its pharmacological effects [25]. Current existing studies have shown that the deglycosylation of PD to resveratrol mainly occurs through two pathways: the first pathway is through intracellular glycosidase lysis, and the second is through membrane-bound enzyme lactase-phlorizin hydrolase. Subsequently, the released glycoside ligands are passively diffused and further metabolized into two glucuronic acid conjugates within the cell. Resveratrol, glucosylated resveratrol and glucosylated PD are the first metabolites of PD in the small intestine and liver of rats. After oral administration of PD, 98.4% of PD was metabolized in the intestine and liver, and the content of resveratrol acidified with glucose aldehyde was as high as 84%, which was the main product of the metabolic reaction [26]. The study of pharmacokinetics also provides ideas for the toxicological research of PD. In a study by Schimith et al. [27], zebrafish were used as a model to investigate the effects of PD on survival, incubation, development and behavior. The results showed that PD at concentrations up to 435 μM exhibited no toxicity and lacked teratogenic, cardiotoxic, and neurotoxic effects. The low toxicity of PD suggests significant potential for related drug development.

### 2.4 Systemic modulation of PD: implications for SCI therapy

**Fig. 1:**
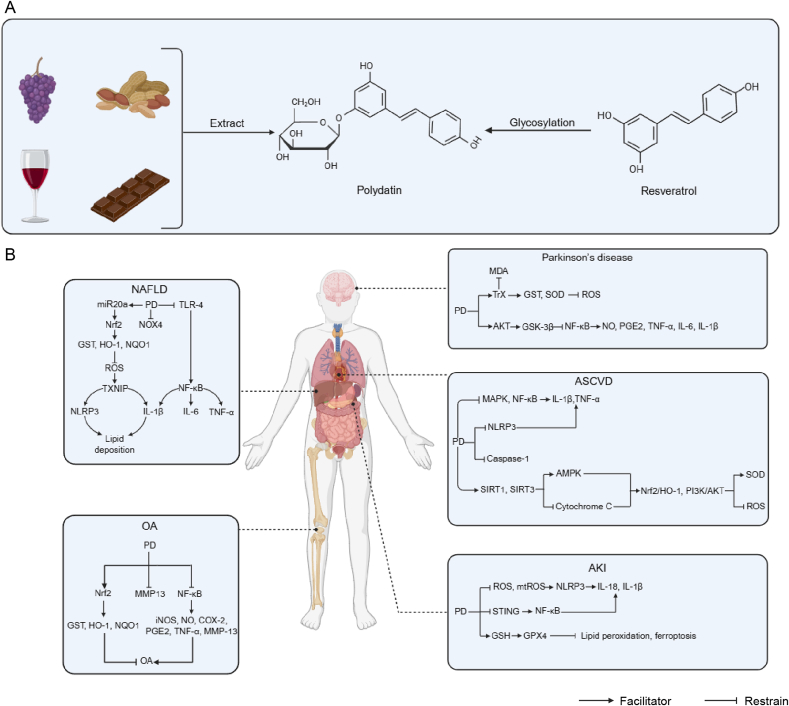
The therapeutic value of the anti-inflammatory, antioxidant, and anti-apoptotic effects of polydatin (PD) in different diseases. (A) Common sources of PD and structural differences with resveratrol. (B) Therapeutic mechanism of PD in multi-system diseases. Non-alcoholic fatty liver disease (NAFLD): PD activates microRNA 20a (miR20a), exerting anti-inflammatory and inhibitory effects on lipid deposition by regulating toll-like receptor-4 (TLR-4). Meanwhile, PD inhibits nicotinamide adenine dinucleotide phosphate hydrogen (NADPH) oxidase 4 (NOX4) enzyme to alleviate oxidative stress. Osteoarthritis (OA): PD alleviates OA-related inflammation by inhibiting the expression of nuclear factor kappa-B (NF-κB) and the production of matrix metalloproteinases-13 (MMP-13), and enhancing the expression of the nuclear factor erythroid 2-related factor 2 (Nrf2) pathway. Parkinson's disease: PD activates thioredoxin (TrX) to reduce oxidative stress, activates the glycogen synthase kinase-3β (GSK-3β) pathway, and inhibits neuroinflammation. Atherosclerotic cardiovascular disease (ASCVD): PD inhibits the inflammatory responses mediated by the nucleotide-binding oligomerization domain (NOD)-like receptor thermal protein domain associated protein 3 (NLRP3) inflammasome pathway, mitogen-activated protein kinase (MAPK) and NF-κB pathways, activates the silent information regulator 1 (SIRT1) antioxidant pathway, and at the same time, PD inhibits the expression of apoptosis-related proteins to combat the pathological changes of ASCVD. Acute kidney injury (AKI): PD inhibits the activation of NLRP3 inflammasome and mitochondrial oxidative stress and suppresses the expression of stimulator of interferon genes (STING) to alleviate kidney injury. This figure integrates the multi-target effects of PD, including common mechanisms such as anti-inflammation, anti-oxidation and anti-apoptosis. GST: glutathione S-transferase; HO-1: heme oxygenase-1; NQO1: nicotinamide adenine dinucleotide (phosphate) quinone oxidoreductase 1; ROS: reactive oxygen species; mtROS: mitochondrial reactive oxygen species; TXNIP: thioredoxin interacting protein; IL: interleukin; TNF-α: tumor necrosis factor-α; iNOS: inducible nitric oxide synthase; COX-2: cyclooxygenase-2; NO: nitric oxide; PGE2: prostaglandin E2; MDA: malondialdehyde; SOD: superoxide dismutase; PI3K: phosphatidylinositol-3 kinase; AKT: protein kinase B; AMPK: adenosine 5’-monophosphate-activated protein kinase; GPX4: glutathione peroxidase 4.

Many studies have proved the pharmacological effects of PD, mainly including anti-inflammation, anti-oxidative stress and anti-apoptosis, and these regulatory effects are also indispensable parts in the treatment process of SCI. The following text will introduce some studies on the therapeutic effects of PD in diseases to inspire us to pay attention to its potential in treating SCI (Fig. 1).

Atherosclerosis is one of the most common diseases in the cardiovascular system. The pathological process includes inflammation, endothelial cell injury, plaque formation, etc. The research by Zhang et al. [28] confirmed that PD can inhibit the activation of nucleotide-binding oligomerization domain (NOD)-like receptors (NLRs) thermal protein domain associated protein 3 (NLRP3) inflammasome and the lysis of caspase-1, thereby suppressing the secretion of inflammatory cytokines and pyroptosis, and alleviating the related symptoms of atherosclerosis. The research by Li et al. [29] found that after injecting PD into mice with non-alcoholic steatohepatitis, oxidative stress could be reduced by downregulating the nicotinamide adenine dinucleotide phosphate hydrogen (NADPH) oxidase 4 (NOX4) enzyme. Furthermore, PD can inhibit the Toll-like receptor-4 (TLR-4)/nuclear factor kappa-B (NF-κB) p65 signaling pathway, thereby reducing inflammation and macrophage activation. Due to its anti-inflammatory, antioxidative, and atmospheric particle oxidation potential-reducing properties, PD can improve respiratory diseases induced by PM2.5 [30]. Renal fibrosis is a key factor for the decline of renal function in patients with chronic kidney disease, mainly due to chronic inflammation of the kidneys. PD reduces the expression of stimulator of interferon genes (STING) protein, thereby inhibiting the activation of downstream targets such as TANK binding kinase 1 (TBK1) phosphorylation and NF-κB nuclear translocation, ultimately suppressing the production of pro-inflammatory factors and improving the pathological changes of renal inflammatory fibrosis [31]. PD mitigates oxidative stress and inflammatory damage by reducing reactive oxygen species (ROS), offering a novel therapeutic strategy for kidney damage caused by oxidative stress and inflammatory responses in kidney stones. In addition, the antioxidant, anti-inflammatory, and inhibiting ferroptosis effects of PD may improve renal function in acute renal injury by enhancing autophagy and restoring silent information regulator 6 (SIRT6)-mediated autophagy flux [32]. PD also protects against gouty nephropathy by downregulating NLRP3, gasdermin D (GSDMD), and caspase-1 protein expression and inhibiting pyroptosis in renal tubular epithelial cells [33]. Tang et al. [34] used interleukin-1β (IL-1β) to induce chondrocytes, causing excessive production of pro-inflammatory mediators within them, and PD treatment could reverse the production of these mediators. In addition, Sun et al. [35] demonstrated that PD glycosides significantly inhibited the transmission of the NF-κB signaling pathway, reversed the M1 polarization of macrophages induced by IL-1β, and thereby treated osteoarthritis lesions.

PD's anti-inflammatory and antioxidant properties may provide neuroprotection in traumatic brain injury [36]. In a Ts65Dn mouse model of Down syndrome, PD restored neurogenesis, neuron number, and dendritic development, improving cognitive function [37]. PD inhibits endoplasmic reticulum stress following subarachnoid hemorrhage, mediated by SIRT1, while reducing neuronal apoptosis and ferroptosis [38]. PD reduces oxidative damage, inflammatory responses, and neuronal apoptosis, improves morphological structure, and decreases cerebral infarction size in rat models of cerebral ischemia-reperfusion injury [39]. In studies related to Parkinson's disease, PD has been found to protect mitochondrial function in the substantia nigra striatum by activating the thioredoxin (Trx) system, restoring adenosine triphosphate (ATP) levels and reducing ROS production [40].

### 2.5 Systemic medication for PD

In clinical practice, the pathophysiological process of SCI patients is complex, usually involving the pathological responses of multiple organs and tissues throughout the body. The research on pharmacokinetics and the therapeutic effects of multi-system diseases in PD provides a basis for the interactions between organs after systemic medication for PD. After systemic administration of PD, it is mainly metabolized through the liver. The liver-protective effect of PD can effectively reduce pro-inflammatory cytokines in the circulation without imposing excessive metabolic burden on the liver [29]. Secondly, the intestinal tract is crucial for the absorption of PD, and the interaction between PD and the intestinal flora ensures the smooth progress of this process, converting it into resveratrol to exert its effect. In the excretion process, the role of PD in treating urinary system diseases such as renal failure indicates that it does not impose excessive burden on the kidney [41]. These studies demonstrate the great potential of PD in the treatment of SCI.

## 3 Pathophysiology of SCI

**Fig. 2:**
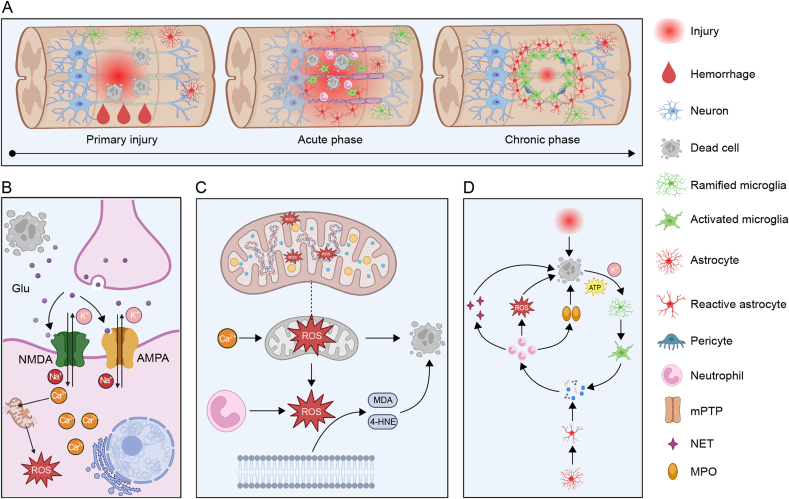
Summary of pathophysiological mechanisms of spinal cord injury (SCI). (A) The figure shows the pathophysiological reactions of primary injury, secondary injury in acute phase, and chronic phase of SCI. The primary injury mainly includes bleeding, cell death, axon rupture and other traumatic events. The acute stage of secondary injury mainly includes the activation of microglia and astrocytes, aggregation of neutrophils, demyelination, apoptosis and other traumatic events. The chronic phase is mainly composed of microglia, astrocytes and pericytes, which form scars and shrink the wound size. (B) The figure shows the mechanism of excitatory toxicity of glutamate (Glu). Damaged cells release Glu to act on amino-3-hydroxy-5-methyl-4-isoxazole-propionic acid receptor (AMPA) and N-methyl-D-aspartic acid receptor (NMDA) in neighboring cells, resulting in the inflow of Ca^2+^ and Na^+^ and the outflow of K^+^. Excessive Ca^2+^ inflow into mitochondria not only leads to the opening of mitochondrial permeability transition pore (mPTP) channels but also leads to the production and release of excess reactive oxygen species (ROS), resulting in a series of damage reactions such as mitochondrial rupture and cell death. (C) The figure shows the mechanism of oxidative stress response after SCI. A sharp increase in Ca^2+^ enters the mitochondria and damages mitochondrial DNA (mtDNA), proteins, and mitochondrial membranes, causing mitochondrial dysfunction and, together with ROS released by neutrophils, causing a large accumulation of free radicals. The polyunsaturated fatty acids in the cell membrane are induced by free radicals to undergo lipid peroxidation to produce 4-hydroxynonenal (4-HNE), malondialdehyde (MDA) and other products, resulting in cell membrane rupture and additional cell death. (D) The figure shows the vicious circle formed by inflammatory response after SCI. In the early stage of injury, the dead cells activate microglia and astrocytes to release inflammatory factors, recruit neutrophils, and produce ROS, neutrophil extracellular traps (NET), myeloperoxidase (MPO), and other destructive substances, which further aggravate apoptosis and form a vicious cycle. ATP: adenosine triphosphate.

The spinal cord mainly consists of neurons and glial cells, among which glial cells account for more and mainly include astrocytes, oligodendrocytes and microglia. The interaction between these cells maintains the normal physiological environment of the spinal cord. After the occurrence of SCI, the interaction between cells is disturbed, resulting in a series of harmful events. From a pathophysiological perspective, SCI can be divided into primary and secondary injuries (Fig. 2).

### 3.1 Primary injury

Primary injuries typically result from mechanical trauma caused by pulling or compression, leading to vertebral fractures and displacement [42]. Within minutes, the spinal cord can swell and occupy the entire diameter of the spinal canal at the injury level, causing secondary ischemia [3]. Direct injury to the spinal cord and subsequent ischemia and hypoxia not only lead to cell death and axon rupture but also initiate a cascade of secondary injuries that continue for days, weeks or even months, leading to further cell death and SCI deterioration.

### 3.2 Secondary injury

Secondary injury can be categorized into three stages based on time: acute, subacute, and chronic. Following the primary injury caused by the initial trauma, the acute secondary injury stage begins, characterized by vascular damage, ion imbalance, excitotoxicity, lipid peroxidation, inflammation, and necrosis. After SCI occurs, ischemia and hypoxia lead to rapid cell death, and damaged cells, axons, and blood vessels release toxic chemicals that harm the integrity of neighboring cells. A cascade of destructive events, including calcium overload due to excitotoxicity, massive production of free radicals, and dysregulated release of Na^+^ and K^+^, ensues. If the acute secondary injury persists, the subacute stage follows, during which a wave of apoptosis sweeps through oligodendrocytes, a form of cell death akin to cellular suicide [43,44]. As an important component of myelin, the death of oligodendrocytes leads to demyelination and slows down signaling along the axon, worsening the condition of axons and oligodendrocytes. In addition, in the early stage of SCI, large amounts of Ca^2+^ flow into axons lead to acute axonal degeneration, which activates cysteine protease and induces Waller's degeneration, further promoting axonal degeneration [45]. As SCI progresses to the chronic secondary injury stage, the most notable feature is the maturation of the glial sar [46]. The glial scar is primarily formed by the proliferation of astrocytes, serving as the body's protective response to initiate healing after SCI [47]. Pericytes and connective tissue also contribute to scar formation, with pericytes secreting specific markers that stimulate fibroblasts to produce extracellular matrix components, such as fibronectin, which are key constituents of scar connective tissue [48].

#### 3.2.1 Glutamate excitatory toxicity and ion imbalance

Glutamate excitatory toxicity is one of the important mechanisms of secondary SCI. Glutamate is the main excitatory neurotransmitter in the brain and spinal cord and is released in small amounts from the ends of axons. Glutamate receptors are widely distributed in the CNS, which can be divided into ionotropic receptors and metabotropic receptors: ionotropic receptors include α-amino-3-hydroxy-5-methyl-4-isoxazole-propionic acid (AMPA) and N-methyl-d-aspartate (NMDA). Glutamate interacts with these receptors, stimulating target cells and generating nerve impulses [49,50]. After SCI, glutamate leakage from damaged spinal cord neurons, axons, and astrocytes, coupled with decreased uptake of glutamate by astrocytes, Na^+^/K^+^ ATPase depletion, and lipid peroxidation, lead to abnormally elevated levels of glutamate [51]. At the same time, glutamate receptors are abnormally activated, causing glutamate to overexcite adjacent neurons, leading to excitatory toxicity and subsequent cell death [52].

Excitatory toxicity is often associated with ion imbalance. Glutamate recognizes AMPA and NMDA receptors on the surface of target cells, resulting in excessive inflow of Na^+^ and Ca^2+^ and excessive outflow of K^+^ from the cell. These changes disrupt cellular ion homeostasis, triggering a series of damaging events such as cytotoxic edema and axonal acidosis [53,54]. Additionally, calcium ion waves released by cells promote the production of free radicals, which are highly reactive molecules that attack cell membranes and other cellular components, leading to neuronal death. Dysregulation of Ca^2+^ concentrations also activates calcium-dependent kinases and phospholipases, resulting in calpain-mediated protein degradation and oxidative damage [55].

Under normal physiological conditions, mitochondria generate sufficient ATP for cellular activities through processes such as the electron transport chain (ETC) and oxidative phosphorylation, which rely on the mitochondrial proton gradient [56]. After SCI, abnormal Ca^2+^ influx activates calcium-induced calcium release from the endoplasmic reticulum, amplifying calcium signaling and causing intracellular calcium overload. Ca^2+^ enters mitochondria via mitochondrial calcium transporters, leading to the opening of mitochondrial permeability transition pores (mPTPs). This disrupts the proton gradient, inhibits ATP production, and causes mitochondrial swelling, ultimately resulting in cell death [57]. Neuronal survival depends on adequate ATP reserves, and mitochondrial dysfunction due to glutamate toxicity can lead to neuronal death [58]. Studies have also shown that excitatory toxicity may contribute to white matter damage and oligodendrocyte death.

#### 3.2.2 Oxidative stress

Under normal conditions, the body's oxidative and antioxidant systems maintain a dynamic balance, ensuring mitochondrial stability. Mitochondria provides essential energy for cellular activities through ETC reactions. In most cells, the ETC consumes 90% of cellular oxygen, with approximately 2% converted into oxygen free radicals within mitochondria [59]. Mitochondria are the primary source of ROS, which mainly exist as O_2_^−^, hydrogen peroxide (H_2_O_2_), and ·OH [60]. Under physiological conditions, mitochondria regulate excessive cytoplasmic calcium levels through an energy-dependent Ca^2+^ buffering system, keeping ROS levels minimal, which is crucial for normal cellular redox reactions [61]. After SCI, demyelination consumes large amounts of ATP, directly impairing the Ca^2+^ buffering system. Ca^2+^ overload further increases ROS production. In rat models of SCI, mitochondrial ROS (mtROS) levels were significantly elevated 4 h post-injury compared to controls [62]. Studies have shown that excessive mtROS production directly damages mitochondrial DNA (mtDNA), proteins, lipids, and other macromolecules, leading to respiratory chain disruption, mitochondrial damage, and autophagy. Beyond mitochondria, phagocytes such as neutrophils and macrophages also contribute to ROS production after SCI. These reactions disrupt local homeostasis, causing excessive accumulation of free radicals and reactive nitrogen species (RNS), and triggering toxic reactions such as lipid peroxidation, DNA damage, and cell death [63].

The spinal cord's high content of polyunsaturated fatty acids makes it particularly susceptible to ROS-induced lipid peroxidation. The reaction of excess free radicals with nitric oxide (NO) after SCI leads to the accumulation of peroxynitrite (PNT), which causes lipid peroxidation of polyunsaturated fatty acids, producing harmful byproducts such as 4-hydroxynonenal (4-HNE), malondialdehyde (MDA), and lipid peroxides, and damaging proteins and DNA. The detailed process involves ROS reacting with polyunsaturated fatty acids in cell membranes, extracting electrons and binding to lipid molecules to form reactive lipid species, which then propagate free radical reactions and eventually produce end products. As a critical component of the blood-spinal cord barrier (BSCB), endothelial cells are tightly connected to prevent large molecules and harmful substances from entering the spinal cord. However, ROS-induced lipid peroxidation significantly promotes endothelial cell death and disrupts the BSCB [64,65]. Functional recovery and tissue repair after SCI largely depend on the integrity of the BSCB.

#### 3.2.3 Inflammation

Under normal conditions, the inflammatory response is the body's defense mechanism against harmful stimuli. When regulated within compensatory limits, it helps combat damage; however, excessive inflammation leads to decompensation and further injury [66]. The normal spinal cord contains various immune cells, including innate immune cells (e.g., peripheral neutrophils, monocytes, and macrophages; CNS astrocytes and microglia) and adaptive immune cells (such as B lymphocytes and T lymphocytes). After SCI, these multicellular interactions are disrupted, breaking the normal physiological homeostasis of the spinal cord [51].

Primary SCI directly causes cell necrosis, ischemia, neuroinflammation, and excitotoxicity, leading to the death of additional neurons and glial cells. The release of ATP, DNA, and potassium activates microglia, which initially adopt a protective phenotype but quickly transition to a pro-inflammatory state, mediating further secondary damage [67,68]. Activated microglia then stimulate astrocytes and peripherally derived macrophages, which amplify inflammation by releasing cytokines and chemokines, promoting sustained apoptosis of neurons and oligodendrocytes [69,70]. Microglia exhibit two main phenotypes: M1, which promotes neuroinflammation, and M2, which suppresses inflammation and supports tissue repair. The balance between these phenotypes is dynamic and influenced by microenvironmental factors, determining their functional roles [71]. Studies have shown that within hours of SCI, spinal cord-resident cells secrete large amounts of inflammatory cytokines, including IL-1α, IL-1β, IL-6, and tumor necrosis factor-α (TNF-α) [67,72]. The production of these cytokines and chemokines, combined with vascular rupture, leads to extensive infiltration of immune cells, which further produce inflammatory mediators [61,73,74]. Neutrophils are the most abundant infiltrating cells in the pro-inflammatory environment early after SCI [75]. Neutrophil levels rise within hours of injury, peaking at 24 h [76]. The presence of neutrophils has both advantages and disadvantages. While neutrophils can penetrate the injury site, guide macrophages to clear debris, and improve the microenvironment, their overactivation by free radicals can have detrimental effects. Neutrophils release neurotoxic substances such as myeloperoxidase (MPO), ROS, RNS, chemokines, and proteases, exacerbating tissue edema and promoting apoptosis of neurons and oligodendrocytes [77]. Additionally, neutrophils produce neutrophil extracellular traps (NETs), which worsen injury by promoting neuroinflammation and disrupting the BSCB [78]. Inflammation driven by microglia and peripherally derived monocyte-derived macrophages (MDMs) peaks around 7 days post-SCI and can persist for months in mice, rats, and humans [67,79]. MDMs also exhibit M1 and M2 phenotypes. Studies have shown that increased intracellular iron and myelin debris regulate macrophage polarization, favoring the pro-inflammatory M1 phenotype [80]. While blood-derived macrophages have greater scavenging capacity than microglia, excessive engulfment of lipid-rich myelin debris can lead to the formation of foam macrophages, reducing phagocytic efficiency and causing further nerve tissue damage [81].

After SCI, lymphocyte counts are reduced. T lymphocytes and B lymphocytes, key players in adaptive immunity, are primarily involved, with CD8^+^ T cells being the dominant subtype. T cell levels peak around 9 days post-SCI [82,83]. Activated by antigen-presenting cells at the injury site, T cells infiltrate the damaged area through the BSCB, contributing to the inflammatory cascade.

## 4 Mechanisms of PD in treating SCI

**Fig. 3:**
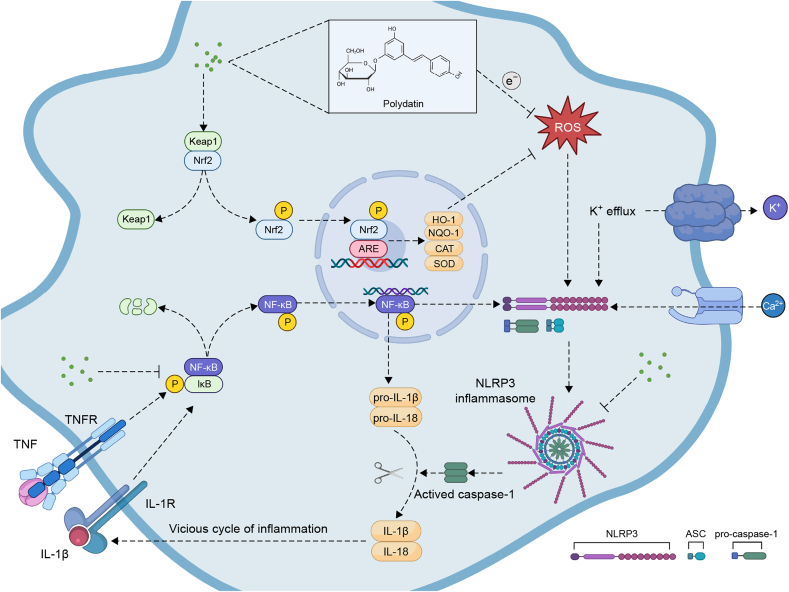
Mechanisms of polydatin (PD) treatment of spinal cord injury (SCI). The figure illustrates the molecular mechanisms by which PD exerts its antioxidant and anti-inflammatory effects after entering cells. The antioxidant effects of PD are manifested both through direct scavenging of reactive oxygen species (ROS) and via activation of the nuclear factor erythroid 2-related factor 2/anti-oxidative response element (Nrf2/ARE) pathway, which enhances the expression of multiple antioxidant genes. Meanwhile, the anti-inflammatory effects of PD are primarily achieved by inhibiting the nuclear factor kappa-B (NF-κB) and nucleotide-binding oligomerization domain (NOD)-like receptor thermal protein domain associated protein 3 (NLRP3) inflammasome pathways, thereby reducing the release of inflammatory factors. Keap1: kelch-like ECH-associated protein 1; HO-1: heme oxygenase-1; NQO1: nicotinamide adenine dinucleotide (phosphate) quinone oxidoreductase 1; SOD: superoxide dismutase; CAT: catalase; IL: interleukin; TNF: tumor necrosis factor; ASC: apoptosis-associated speck-like protein; IκB: inhibitor of NF-κB.

Studies have demonstrated that PD exerts significant therapeutic effects following SCI, including the restoration of motor and sensory functions, alleviation of oxidative stress, inhibition of apoptosis, reduction of neuroinflammation, and provision of neuroprotection. Below is a review of relevant research and findings on the role of PD in treating SCI. In these studies, the source of PD was all purchased powdered PD (purity ≥ 95%), which was dissolved in DMSO and diluted to the required concentration with normal saline or culture medium before conducting *in vivo* or *in vitro* experiments (Table 1 [[84], [85], [86], [87], [88]] and Fig. 3).

**Table 1:** Summary of animal and cell experiments of polydatin (PD) in the treatment of spinal cord injury (SCI).

Type of experiment	Experimental model	Experimental group	Results	Refs.
Animal experiment	Male rats spinal cord weight-drop model	● Sham ● Control ● Treatment-L ● Treatment-H	BBB↑ Wet/dry weight ratio↓ Nrf2↑, HO-1↑ SOD↑, MDA↓ Cleaved caspase-3↓ Bax↓, Bcl-2↑	[84]
	Male rats aneurysm clip compression model	● Sham ● Control ● Treatment-L ● Treatment-M ● Treatment-H	BBB↑ Pain threshold↑ Nitrite↓ CAT↑, GSH↑ MMP-2↑ Number of neurons↑	[86]
	Male rats spinal cord weight-drop model	● Sham ● Control ● Treatment-L ● Treatment-H	BBB↑ Wet/dry weight ratio↓, iNOS↓ NO↓ IL-1β↓, IL-6↓, TNF-α↓	[88]
	Mice pneumatic impact device compression model	● Sham ● Control ● Treatment-I (PD) ● Treatment-II (BMSCs) ● Treatment-III (PD + BMSCs)	BBB↑ Wet/dry weight ratio↓ MAP-2↑ GFAP↓, Glial scar↓ Number of neurons↑	[87]
Cell experiment	LPS stimulates BV2 cells	–	ROS↓ LDH↓ Nrf2↑, HO-1↑ Cleaved caspase-3↓ Bax↓, Bcl-2↑	[84]
	H_2_O_2_ stimulates BMSCs	–	Cleaved caspase-3↓ Bax↓, Bcl-2↑ ROS↓ LDH↓, GSH↑ Nrf2↑, NQO1↑	[85]
	LPS stimulates BV2 cells	–	IL-1β↓, IL-6↓, TNF-α↓ NF-κB↓ iNOS↓NO↓ NLPR3 Inflammasome↓	[88]
	–	● Blank Control (BMSCs) ● BMSCs + PD	MAP-2↑, NeuN↑ NF-M↑, NSE↑ ChAT↑ Nrf2↑, NQO1↑, HO-1↑	[87]

MAP-2, NeuN, NF-M, ChAT and NSE are markers of neural differentiation. ↑/↓ indicates that the indicator goes up or down. Sham: sham operation group; Treatment-L/M/H: low, medium and high dose PD treatment group; BBB: Basso-Beattie-Bresnahan scoring test; Nrf2: nuclear factor erythroid 2-related factor 2; HO-1:heme oxygenase-1; SOD: superoxide dismutase; MDA: malondialdehyde (marker of lipid peroxidation); Bcl-2: B-cell lymphoma-2; Bax: Bcl-2-associated X protein; CAT: catalase; GSH: glutathione; MMP-2: matrix metalloproteinase-2; iNOS: inducible nitric oxide synthase; IL: interleukin; TNF-α: tumor necrosis factor-α; MAP-2: microtubule-associated protein 2; GAFP: glial fibrillary acidic protein; –: no data; LDH: lactate dehydrogenase; NQO1: nicotinamide adenine dinucleotide (phosphate) quinone dehydrogenase 1; NF-κB: nuclear factor-kappa B; BMSCs: bone marrow stem cells ; ChAT: choline acetyltransferase; NeuN: neuronal nuclear antigen; NF-M: neurofilament medium; NSE: neuron-specific enolase.

### 4.1 Restoring motor and sensory function

Following SCI, a cascade of injury responses can severely impair motor and sensory functions. In rat models of SCI, the primary motor and sensory impairments observed include hindlimb paralysis, gait abnormalities, muscle atrophy, reflex dysfunction, and paresthesia. These impairments are attributed to disruptions in neural and sensory pathways, neuronal death, and inflammatory responses. The functions of PD including anti-inflammatory, antioxidant, and anti-apoptotic properties play a pivotal role in mitigating neurodegeneration. Consequently, numerous studies have explored the therapeutic potential of PD in restoring motor and sensory functions in SCI rats. This can provide a macroscopic basis for the effect of PD in treating SCI.

#### 4.1.1 Recovery of motor function

The Basso-Beattie-Bresnahan (BBB) scoring test is a widely used standard for evaluating behavioral function in experimental rats. This test, developed in 1995 based on the Tarlov open-field method, involves observing hindlimb movements for 4 min and scoring them on a scale from 0 (no movement) to 21 (normal movement), with the average score of both hindlimbs serving as the result. Additionally, the inclined plane test is commonly used to assess motor function post-SCI. In this test, rats are placed on an adjustable inclined plane, and the maximum angle at which the animal maintains balance for over 5 s is recorded as the score [89]. Studies have shown that the BBB scores of rats with SCI are significantly lower than those of sham-operated rats (laminectomy only). However, intraperitoneal injection of 20 or 40 mg/kg PD treatment improves BBB scores within 14 days post-surgery. Furthermore, by comparing the spinal cord wet/dry weight ratios across groups, PD treatment has been shown to significantly alleviate spinal cord congestion, edema, and structural damage in SCI rats, which is critical for motor function recovery [84]. Bavandpouri et al. [86] found that compared with the SCI group, rats treated with intravenous injection of 2 mg/kg PD had higher scores in BBB and slant tests, and showed significant improvements in weight-bearing capacity and gait coordination, almost reaching the functional level of the pseudo-operation group. On this basis, Zhan et al. [87] conducted electrophysiological analysis and found that SCI significantly reduced the amplitude of spinal cord evoked potential (SCEP) and prolonged the latency of SCEP, while PD treatment significantly increased the amplitude of SCEP (0.73 ± 0.08 mv) and shortened the latency of SCEP (5.45 ± 0.57 ms). These findings collectively demonstrate PD's efficacy in restoring motor function post-SCI.

#### 4.1.2 Recovery of sensory function

Sensory dysfunction following SCI primarily includes partial or complete loss of sensation below the injury level, hyperalgesia, and proprioceptive disturbances. Experimental methods for assessing sensory dysfunction in animals have been well-documented. For instance, cold allodynia can be evaluated by spraying acetone onto the plantar surface of the hindpaws and observing withdrawal responses and reaction times. Heat hyperalgesia is assessed using a hot plate apparatus, recording the latency to paw licking or jumping. Mechanical allodynia is tested using von Frey filaments applied to the lateral plantar area of the hindpaw, with abnormal pain responses indicating sensory dysfunction [90,91]. In experiments conducted by Bavandpouri et al. [86], analysis of variance of cold and heat pain response thresholds revealed that SCI rats exhibited significant hypersensitivity to both stimuli compared to the sham group. However, PD treatment increased the response thresholds to cold and heat stimuli in injured animals. Additionally, SCI caused a sustained reduction in paw withdrawal thresholds, indicating mechanical allodynia, which was effectively alleviated by PD treatment starting from day 7 post-injury. These results suggest that PD can mitigate sensory dysfunction following SCI.

### 4.2 Antioxidant effects

**Fig. 4:**
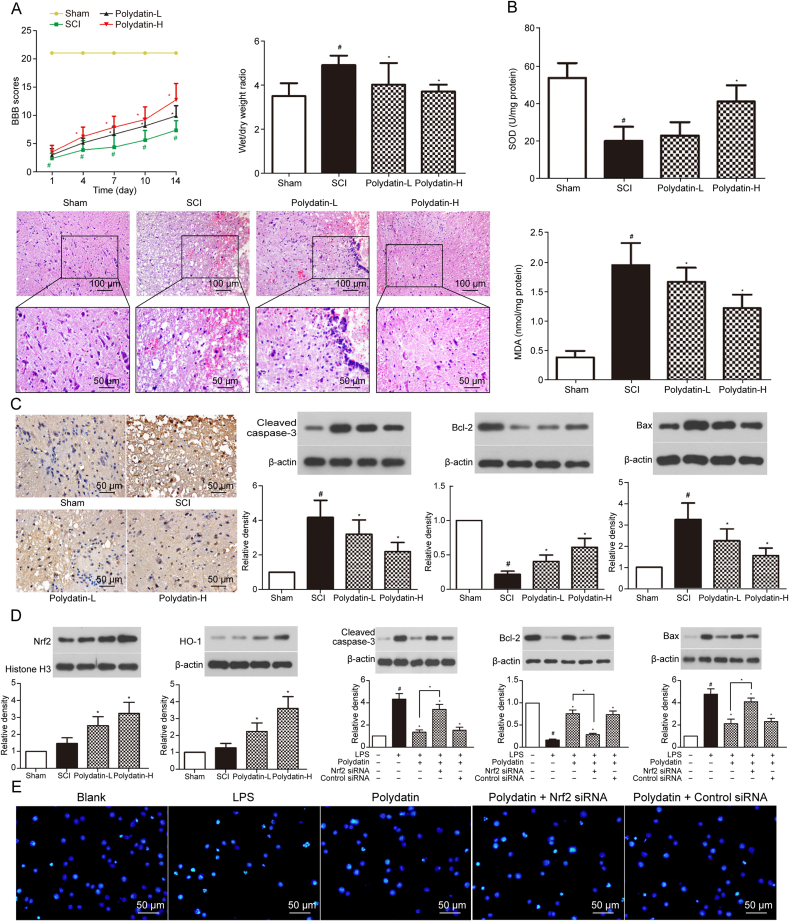
Polydatin (PD) attenuates spinal cord injury (SCI) in rats by inhibiting oxidative stress and microglia apoptosis via nuclear factor erythroid 2-related factor 2/heme oxygenase-1 (Nrf2/HO-1) pathway. (A) The improvement effect of PD on the Basso-Beattie-Bresnahan (BBB) scoring test after SCI, the dry/wet ratio of spinal cord, and the morphology of spinal cord tissue. (B) The release of superoxide dismutase (SOD) and malondialdehyde (MDA) before and after the action of PD. (C) The effect of PD on cell apoptosis: terminal deoxynucleotidyl transferase dUTP nick end labeling (TUNEL) staining, Western blot. (D) Expression of Nrf2/HO-1 pathway-related proteins and apoptosis-related markers. (E) The inhibitory effect of PD on apoptosis of BV2 cells. Reprint from Ref. [84] with permission.

The antioxidant capacity of PD has been well-documented. On the one hand, the phenolic hydroxyl groups in PD can directly neutralize ROS, reducing oxidative damage to proteins and DNA, thereby protecting neurons and glial cells [9]. On the other hand, PD modulates key signaling pathways associated with oxidative stress and apoptosis, demonstrating significant biological activity [92]. Additionally, studies have shown that PD protects mitochondria by inhibiting the release of cytochrome *c* and regulating the SIRT3/superoxide dismutase 2 (SOD2) pathway, thereby reducing oxidative damage linked to neurodegeneration [93] (Fig. 4) [84].

#### 4.2.1 Nrf2/ARE signaling pathway

Studies have confirmed that PD activates the nuclear factor erythroid 2-related factor 2/anti-oxidative response element (Nrf2/ARE) signaling pathway, promoting intracellular antioxidative stress responses and exerting cytoprotective effect [94]. Nrf2 is a leucine zipper transcription factor that regulates molecules related to oxidative stress, such as SOD, ROS, and glutathione (GSH), playing a crucial role in maintaining cellular redox homeostasis [95]. Under physiological conditions, Nrf2 binds to Kelch-like ECH-associated protein 1 (Keap1) in the cytoplasm, and its activity is negatively regulated by Keap1. When cells are exposed to oxidative stress or chemical stimuli, Nrf2 dissociates from Keap1 and translocates to the nucleus, where it regulates the transcription of antioxidant genes [96]. ARE is a specific regulatory sequence. Upon binding to ARE, Nrf2 enhances the expression of antioxidant enzymes such as heme oxygenase-1 (HO-1), nicotinamide adenine dinucleotide (phosphate)quinone oxidoreductase 1 (NQO1), glutathione peroxidase (GPX), and SOD, thereby reducing oxidative damage [97]. Relevant studies have reported that Nrf2 knockout in the nervous system of mice exacerbates spinal cord tissue damage in SCI models compared to controls, suggesting that PD may inhibit oxidative stress by activating the Nrf2 pathway in injured spinal cord tissue [98].

Lv et al. [84] found that PD treatment could up-regulate the activity of SOD and down-regulate the level of MDA in spinal cord tissue, and the measurement of oxidative stress markers proved that PD could alleviate SCI induced oxidative damage. In addition, in the spinal cord tissues of SCI model rats, the expressions of apoptosis-related proteins including cleaved caspase-3 and B-cell lymphoma-2 (Bcl-2) associated X protein (Bax) were significantly increased, and the expression of Bcl-2 was significantly decreased, while PD treatment almost restored these indicators to normal levels, suggesting the anti-apoptotic ability of PD. As for the specific mechanism, this study found that the levels of nuclear Nrf2 and cytoplasmic HO-1 in spinal cord tissue of PD rats were significantly higher than those in the sham operation group, while the production of ROS and lactate dehydrogenase (LDH) was reduced, suggesting that PD may alleviate oxidative stress and subsequent apoptosis of spinal cord tissue through the Nrf2/HO-1 pathway. To support this idea, the researchers conducted a series of *in vitro* experiments. First, BV2 cells were transfected with Nrf2-specific small interfering RNA (siRNA) to successfully knock out Nrf2 in BV2 cells. Consistent with the experimental results *in vivo*, Nrf2 activity increased after PD treatment in lipopolysaccharide (LPS)-stimulated BV2 cells, and this effect disappeared with the elimination of Nrf2. This fully proves that PD alleviates oxidative stress and a series of apoptotic cascades in spinal cord tissue after SCI by activating Nrf2/HO-1 pathway. Similarly, Bavandpouri et al. [86] demonstrated PD's therapeutic effects on oxidative stress by measuring catalase and GSH levels in SCI rats.

Bone marrow stem cells (BMSCs) possess self-renewal and multilineage differentiation potential, making them promising for treating CNS diseases like SCI. However, their survival is compromised in the oxidative stress environment following SCI [99]. In this study, H_2_O_2_ at a concentration of 600 μM was used to induce oxidative damage in BMSCs, and oxidative stress-related indicators were detected through a series of methods to prove the antioxidant capacity of PD. ROS probe detection showed that compared with the control group, ROS in the H_2_O_2_ treatment group was significantly increased and intracellular GSH was decreased, while these indicators in the PD treatment group were close to normal, suggesting that PD has antioxidant effects. Regarding the relevant mechanism, the experiment found that H_2_O_2_ down-regulates the expression of Nrf2 and NQO1, and PD can be partially reversed. To further support this view, brusatol is used as an Nrf2 pathway inhibitor, selectively downregulating Nrf2 protein levels by increasing Nrf2 ubiquitination and degradation. Under the condition of co-incubation of PD and brusatol, the toxic effect of H_2_O_2_ on cells was remanifested, fully indicating that PD has an antioxidant effect on BMSCs through the Nrf2/NQO1 pathway. More notably, since relevant studies have proved that PD can limit the proliferation of tumor cells by stopping cells at a certain part of the cell cycle, this study found that a concentration of 30 μM of PD had no inhibitory effect on the proliferation of BMSCs. In conclusion, PD not only protects BMSCs from oxidative damage and death, but also does not cause cell cycle arrest. This is a very promising method to improve the cell survival rate of cell replacement therapy in SCI [85].

#### 4.2.2 Regulating iNOS activity and NO levels

Under normal physiological conditions, NO is present in low concentrations in the CNS as a gaseous chemical messenger, playing roles in synaptic plasticity, receptor function, and neurotransmitter release. However, in the pathological state following SCI, inducible nitric oxide synthase (iNOS) is highly expressed in microglia and vascular smooth muscle cells, leading to excessive NO production and triggering a cascade of inflammatory reactions and oxidative damage [[100], [101], [102]]. Matrix metalloproteinases (MMPs) are a family of zinc-dependent enzymes involved in the breakdown of extracellular matrix proteins during tissue remodeling. Among them, MMP-2 primarily degrades collagen, gelatin, and elastin, with its gene expression regulated during vascular remodeling, injury, and inflammation [103,104]. Studies have shown that inhibiting iNOS can increase MMP-2 activity [105,106]. MMP-9 plays a significant role in blood-brain barrier dysfunction after CNS injury, with its activity increasing during the acute phase of SCI, contributing to glial scarring and neuronal damage [107]. Therefore, targeting the inhibition of iNOS expression and NO production, as well as their effects on MMPs, represents a potential therapeutic strategy for SCI.

In the study by Lv et al. [88], iNOS expression was found to be elevated at both the protein and messenger RNA (mRNA) levels after SCI, accompanied by increased NO levels in spinal cord tissue, consistent with the concept of iNOS overexpression and NO imbalance. PD treatment significantly downregulated iNOS expression and reduced NO levels. Similarly, in LPS-stimulated BV2 cells, PD reversed the increased iNOS expression and NO release induced by LPS. In the study by Bavandpouri et al. [86], SCI rats exhibited elevated nitrite levels and MMP-9 activity, along with reduced MMP-2 activity, compared to the sham group. PD treatment reversed these changes, demonstrating its ability to inhibit iNOS expression, reduce NO production, and regulate MMP activity, thereby alleviating inflammatory damage.

### 4.3 Anti-inflammatory effects

**Fig. 5:**
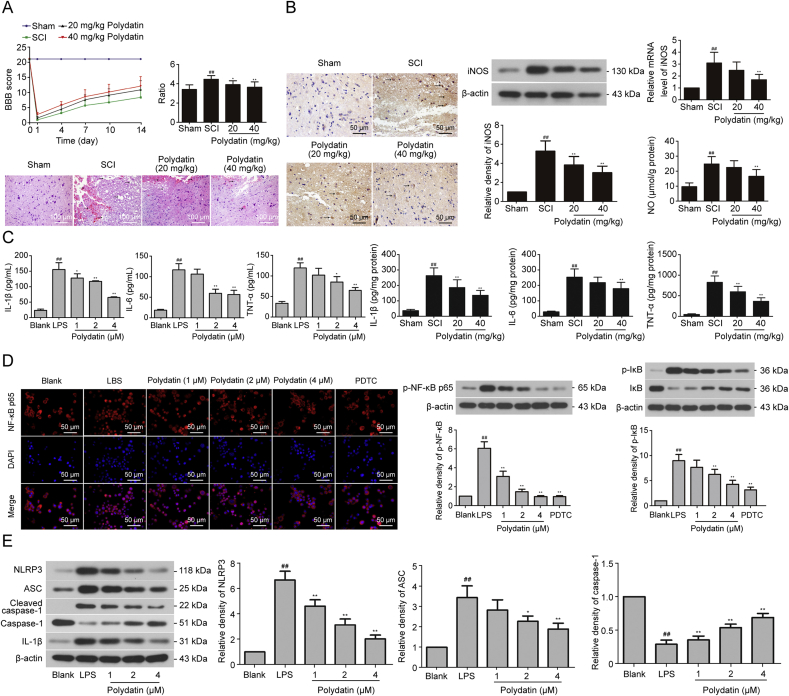
Polydatin (PD) alleviates traumatic spinal cord injury (SCI) by reducing microglial inflammation via regulation of inducible nitric oxide synthase (iNOS) and nucleotide-binding oligomerization domain (NOD)-like receptor thermal protein domain associated protein 3 (NLRP3) inflammasome pathway. (A) The improvement effect of PD on the Basso-Beattie-Bresnahan (BBB) scoring test after SCI, the dry/wet ratio of spinal cord, and the morphology of spinal cord tissue. (B) Effects of PD on NO generation in SCI rats. (C) Effects of PD on interleukin-1β (IL-1β), IL-6 and tumor necrosis factor-α (TNF-α) production. (D) Effects of PD on nuclear factor kappa-B (NF-κB) activation in lipopolysaccharide (LPS)-stimulated BV2 microglia. (E) Effects of PD on NO production in LPS-stimulated BV2 microglia. Reprint from Ref. [88] with permission.

The anti-inflammatory effect of PD has been demonstrated in neurodegenerative diseases by inhibiting NF-κB and blocking the production of intercellular adhesion molecule-1 (ICAM-1) protein/mRNA. Proinflammatory cytokines such as IL-1β, TNF-α, and IL-6 are also reduced by downregulating TLR-2 and NF-κB p65 pathway expression [108]. PD treatment directly reduces inflammatory mediators after SCI and inhibits microglia-induced inflammation by modulating inflammation-related pathways, thereby alleviating inflammatory damage (Fig. 5) [88].

#### 4.3.1 NLRP3 inflammasome pathway

Inflammasome is a protein complex that activates caspase-1 in response to microbial invasion or injury signals. It consists of NLRs, the adapter protein ASC (apoptosis-associated speck-like protein containing a caspase recruitment domain), and caspase-1 [109]. NLRP3, an NLR with a pyrin domain (PYD) at the N-terminus, interacts with ASC via PYD, while ASC binds to caspase-1 through its C-terminal caspase recruitment domain (CARD), promoting caspase-1 activation [110]. The NLRP3 inflammasome pathway consists of two main steps: initiation (Signal 1) and assembly (signal 2). Initiation depends on the NF-κB signaling pathway. When subjected to various internal and external environmental stimuli, the inhibitor of NF-κB (IκB) protein bound to NF-κB is phosphorylated and degraded, so that NF-κB is released and transferred to the nucleus to mediate NLRP3 and inactive IL-1β, IL-18 transcription. The assembly stage is driven by events such as mitochondrial dysfunction, ROS production, K^+^ efflux and Ca^2+^ inflow, which generate NLRP3 inflammasome and then activate caspase-1 to make inactive IL-1β and IL-18 active by cutting [111]. The NLRP3 inflammasome pathway also mediates pyroptosis in inflammatory environments [112]. After SCI, excessive inflammatory factors, including macrophage migration inhibitory factor (MIF), IL-1, IL-6, and TNF-α, accumulate around spinal cord tissue, contributing to secondary injury. Limiting these inflammatory mediators is a key therapeutic strategy for SCI [113]. After SCI, the activation of the NLRP3 inflammasome pathway and the NF-κB signaling pathway, excessive production of inflammatory factors, together with the oxidative stress and ion imbalance environment in the spinal cord tissue, these harmful processes are interlinked and constitute a complex reaction of secondary injury.

In the study of Lv et al. [88], it was found that the levels of pro-inflammatory factors such as IL-1β, IL-6 and TNF-α in the spinal cord of rats after SCI were significantly increased, and the injection of PD could significantly reduce the concentration of pro-inflammatory factors. Then, LPS stimulation of BV2 led to increased expression of NLPR3 and its related proteins ASC, caspase-1 and IL-1β in microglia. PD treatment significantly inhibited the expression of these proteins. More, the researchers found that LPS stimulation significantly increased the fluorescence intensity of NF-κB p65 in BV2 cells, while PD treatment could reverse this phenomenon, and the phosphorylation level of NF-κB p65 decreased after PD incubation. Importantly, PD was found to act in the same way as pyrrolidine dithiocarbamate (PDTC), a classical NF-κB inhibitor. These results indicate that PD plays a protective role in alleviating post-SCI inflammation by inhibiting the NLRP3 inflammasome pathway, inhibiting NF-κB activation and reducing the levels of pro-inflammatory factors.

### 4.4 Anti-apoptotic effects

**Fig. 6:**
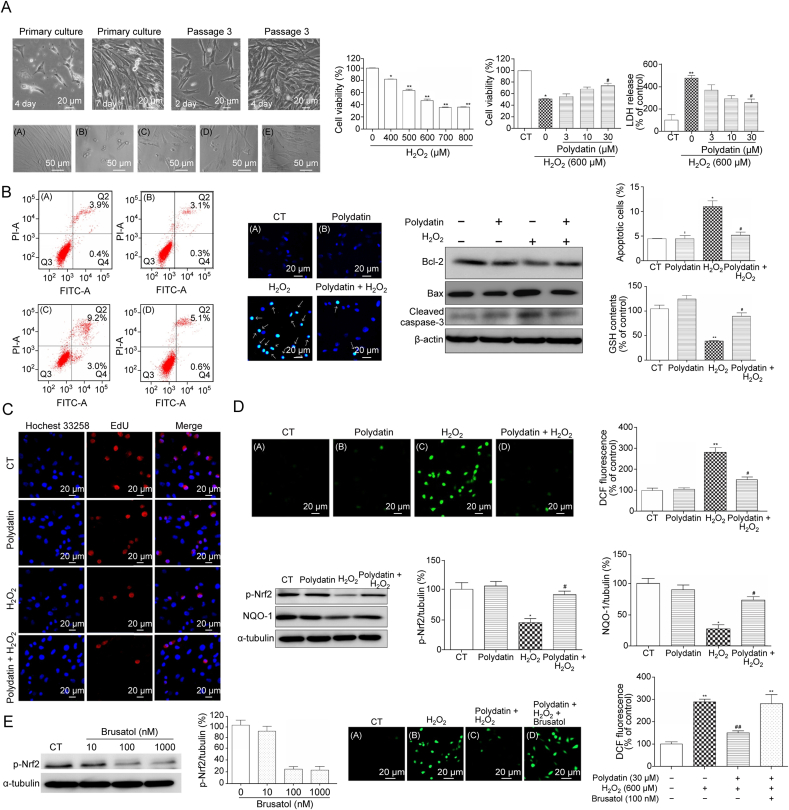
Polydatin (PD) protects bone marrow stem cells (BMSCs) against oxidative injury: involvement of nuclear factor erythroid 2-related factor 2/anti-oxidative response element (Nrf2/ARE) pathways. (A) Representative fields of BMSCs morphologies and effects of PD on the cell viability of BMSCs exposed to hydrogen peroxide (H_2_O_2_). (B) PD attenuated H_2_O_2_-induced apoptosis in BMSCs. (C) PD did not inhibit the proliferation of BMSCs. (D) PD scavenges reactive oxygen species (ROS) produced by H_2_O_2_. (E) PD protected BMSCs against H_2_O_2_-induced cell death partly through Nrf2/ARE pathway. Reprint from Ref. [85] with permission.

A series of cascade reactions caused by secondary injury can form a vicious cycle and aggravate the damage of spinal cord tissue. During these processes, various types of apoptosis persist. Existing studies have shown that PD can regulate apoptosis-related signaling pathways and inhibit apoptosis (Fig. 6) [85].

#### 4.4.1 Inhibit microglial cell apoptosis

Bcl-2 and Bax, members of the Bcl-2 family, are key regulators of apoptosis. Bax induces mitochondrial membrane depolarization and promotes apoptosis, while Bcl-2 inhibits apoptosis by preventing cytochrome *c* release into the cytoplasm. The balance between Bcl-2 and Bax is critical for mitochondrial stability [114]. Additionally, caspase-3, a protease that promotes apoptosis, triggers protein cleavage and DNA fragmentation. The expression of apoptosis-related proteins can be used to explore the mechanisms of apoptosis protection [115].

In the study of Lv et al. [84], it was found that compared with the sham operation group, SCI surgery significantly increased terminal deoxynucleotidyl transferase dUTP nick end labeling (TUNEL)-positive cells in the spinal cord of rats, and the results of TUNEL staining showed that intraperitoneal injection of 20 or 40 mg/kg PD treatment could significantly inhibit cell apoptosis. In addition, in the spinal cord tissues of SCI model rats, the expressions of apoptosis-related proteins were significantly increased, cleaved caspase-3 and Bax, and the expression of Bcl-2 was significantly decreased, while PD treatment almost restored these indicators to normal levels. *In vitro* experiments, researchers treated with LPS significantly increased the apoptosis of BV2 cells, while 4 μM PD treatment could significantly reduce cleaved caspase-3 and Bax and increase the level of Bcl-2. Combined with the above text, PD may play a role in inhibiting microglial apoptosis by suppressing oxidative stress through the Nrf2/ARE pathway.

#### 4.4.2 Inhibit BMSCs apoptosis

BMSCs transplantation is recognized as an effective method for treating SCI. However, due to the complex microenvironment after SCI, the apoptosis rate of BMSCs is very high. Relevant studies have proved that PD can effectively alleviate the apoptosis of BMSCs. Chen et al. [85] used Hochest 33258 staining and Annexin V-propidium iodization (PI) staining to observe whether H_2_O_2_ induced apoptosis. The results showed that PD could significantly reduce apoptosis caused by H_2_O_2_. Furthermore, experimental observations revealed that after H_2_O_2_ treatment, the nuclei of BMSCs condensed, Bax and cleaved caspase-3 were upregulated, and Bcl-2 was downregulated. These apoptosis-related reactions can be reversed under PD treatment, suggesting that PD has an anti-apoptotic effect on BMSCs.

### 4.5 Neuroprotective effects

Neuronal apoptosis, axonal degeneration, and demyelination following SCI are major contributors to spinal cord dysfunction. Although the CNS has limited capacity for axonal regeneration, various cellular and molecular therapies have shown neuroprotective potential in animal models by overcoming the hostile post-injury environment or promoting pathways involved in axon regeneration, neuron survival, and synaptic plasticity [44].

#### 4.5.1 L1 small molecule agonist

Recognition molecules have the potential to promote axon regeneration, neuron survival, and synaptic plasticity. L1, a cell adhesion molecule, plays multiple roles in neurological diseases, including promoting neurite growth, myelination, motor recovery after SCI in rats, and supporting corticospinal tract axon regeneration in adult mice [116,117]. In the study by Kataria et al. [118], PD was identified as one of eight L1 small molecule agonists from a compound library, capable of interacting with L1 and mediating neuroprotective effects. *In vitro* experiments demonstrated that PD stimulates neuron survival, migration, axon growth, Schwann cell proliferation, migration, and myelination. *In vivo* experiments further confirmed that PD improved Basso Mouse Scale (BMS) scores, plantar stepping ability, hip height index, and overall recovery in SCI mice, while reducing inflammatory cell activation and increasing synaptic terminals around motor neurons. These findings highlight PD's potential as an L1 small molecule agonist for neuroprotection after SCI.

#### 4.5.2 Other neuroprotective effects of PD

Numerous studies have demonstrated PD's neuroprotective benefits. PD increases the expression of brain-derived neurotrophic factor (BDNF), which is beneficial in various neurodegenerative diseases [119]. Additionally, PD enhances the survival rate of nerve cells post-injury, reduces cyclin-dependent kinase 5expression, and alleviates neurological deficits [120]. PD also modulates the p38 mitogen-activated protein kinase (MAPK) signaling pathway and mitochondrial apoptosis pathways involving caspase-3/9, improving neurological scores and survival time in brain-injured rats [121]. In the study by Zhan et al. [87], PD mitigated SCI-induced damage to dorsal white matter and central gray matter, reduced motor neuron loss, and promoted axon regeneration while weakening glial scars. Combined with its antioxidative, anti-apoptotic, and anti-neuroinflammatory properties, PD demonstrates excellent potential for functional recovery after SCI.

### 4.6 Other effects of PD in treating SCI

Stem cell transplantation is a novel strategy for addressing nerve damage in SCI. Due to the limited number of resident nerve cells, transplanting BMSCs and promoting their differentiation into neuron-like cells is a promising approach. In the study by Zhan et al. [87], PD combined with BMSCs significantly increased the expression of neuronal markers such as microtubule-associated protein (MAP), neuronal nuclear antigen (NeuN), and neuron-specific enolase (NSE), demonstrating PD's ability to enhance neural differentiation of BMSCs.

Chronic SCI is often accompanied by extensive bone loss below the injury level, particularly in the distal femur and proximal tibia, increasing the risk of osteoporosis and fragility fractures [122]. Zhan et al. [123] studied the therapeutic effect of PD on bone loss in SCI mice by constructing a chronic SCI mouse model. The experimental results showed that PD could restore bone mineral density and bone structure, restore Wnt/β-catenin signaling pathway, and reduce bone loss in chronic SCI mice by regulating the differentiation and activity related genes of osteoblasts and osteoclasts.

## 5 Challenges and prospects

CNS diseases represented by SCI have always been a major problem in the medical field. Thanks to the painstaking research of countless scientists, more and more advanced treatment methods have emerged one after another. The fascinating research achievements such as stem cell therapy, biomaterial scaffolds, electrical stimulation and gene therapy have largely advanced human understanding of CNS diseases. Among them, stem cell therapy represented by MSCs has stood out in recent years, including extensive research on its anti-inflammatory activity and mechanism, as well as successful practices in the combined treatment of neuroinflammation with nanostructured liposomes [[124], [125], [126]]. However, these emerging alternative treatment methods have relatively high costs and complexities. In addition, the safety of long-term use of these treatment measures has not been proven yet, and more clinical trials are needed [127]. In contrast, the method of extracting active ingredients from natural Chinese herbal medicines is simple, safe and economical. This gives us more interest in exploring the possible role that PD may play in the treatment of SCI. Because of this, more challenges and problems to be solved urgently await us.

### 5.1 New drug delivery systems of PD

As mentioned above, compared with resveratrol, the presence of glucoside bonds in PD improves the bioavailability of PD, and the intestinal absorption of PD is higher than that of resveratrol, which brings more possibilities for the application of PD [10]. However, although it is better than resveratrol, the solubility and first-pass metabolism of PD are still unsatisfactory, which limits its full drug effect.

To address these issues, people are exploring new drug delivery systems, including the use of novel materials, chemical structure modification of drugs, and metabolic regulation strategies.

The unique glucose group in PD also opens up possibilities for the development of various nano-preparations, liposomes, micelles (MCs) [128], quantum dots and other delivery systems, thereby enhancing the pharmacodynamics and pharmacokinetics of PD. Studies have shown that PD-loaded chitosan nanoparticles (PD-CSNPs) alleviate diabetic liver injury through different mechanisms, including anti-inflammatory and antioxidant effects as well as influences on carbohydrate metabolic enzymes. PD-CSNPS have higher bioavailability and longer release patterns than free PD. These pieces of evidence are sufficient to prove the advantages of this nanomaterial loaded PD [129]. Some studies have developed liposome systems loaded with PD using thin film hydration technology, which can effectively enhance the fat solubility and promote intestinal absorption. The pharmacokinetic study showed that the relative oral bioavailability of the drug-carrying liposome system was 282.9%, which was significantly higher than that of free drugs [130]. In addition, the dispersion of PD with hydrophilic carrier, such as polyvinylpyrrolidone (PVP) to form an amorphous form can improve the dissolution rate of the drug. In order to treat the intestinal toxicity associated with oxaliplatin in the treatment of colon cancer, PVP-PD nanoparticles with small particle size and slow release performance were prepared using PVP as carrier, which solved the problem of poor water solubility of PD [131]. Also using material PVP, M. Paczkowska-Walendowska et al. [132] found that PVP/hydroxypropyl-β-cyclodextrin (HP-β-CD)-based electrospinning nanofiber materials can release PD completely and for a long time in Polygonum polygonatum. Tablets and powders made from PVP/hpbetacd nanofibers can remain on mucous membranes for longer periods of time, highlighting the particular benefits of combining the new material with PD as a drug delivery system. Exosomes have become a research hotspot in recent years due to their wide range, stable properties, and various types of molecules and drugs that can be encapsulated. Chen et al. [133] successfully studied the preparation of exosome coated PD nanoparticles (exo-PD) to improve the water solubility and bioavailability of PD, and at the same time improve mitochondrial function and reduce radiation damage to intestinal cells. Recyclable nanostructures have potential for drug delivery and metabolism *in vivo*. In this way, PD nanoparticles injected daily through gastric cannula for about a month can enhance the efficacy of PD in the treatment of albino rats [134]. Lin et al. [128] designed PD-loaded MC (PD-MC) based on the ROS and pH dual-sensitive block polymer poly (ethylene glycol)-b-poly (PBEM-co-DPA) and evaluated its effect on liver fibrosis. PD-MC enhances the biocompatibility of PD and improves the release of drugs in the liver in response to the fibrotic microenvironment. Polylactic acid - glycolic acid (PLGA) microspheres loaded with PD exhibit the efficacy of inhibiting lipid peroxidation and oxidative stress, which play a preventive role in oral squamous cell carcinoma [135].

Some studies have shown that the combination of natural products and drugs can significantly improve the effectiveness of treatment. Yang et al. [136] found that Guizhi had a strong influence on the pharmacokinetic parameters and tissue distribution characteristics of PD and could be combined with PD to improve bioavailability. Studies have proved that PD has a more efficient biotransformation ability with the help of probiotics [137,138]. Serafino et al. [139] pointed out in their article that curcumin and PD have synergistic effects on each other, and the combination of the two can improve the efficacy of anti-tumor drugs such as temozolomide on glioblastoma resistant cells. In addition, the glycosidic bond of PD has structural modification potential such as methylation and acetylation, while the hydroxyl group has the modification potential of esterification or etherification, which may play a key role in improving the structural stability.

Whether it is material-loaded PD or drug combination therapy strategies, the novel drug delivery systems used in combination with PD have shown powerful effects in terms of bioavailability and pharmacokinetics, making them highly worthy of further study. However, it is a pity that the PD currently used in the treatment of SCI is all dissolved free PD. There is no PD in the form of combination with new materials or drugs for SCI. We hope that in the future, there will be scientists interested in this field to fill the gap in this area.

### 5.2 Clinical trials and commercialization of PD

PD, as a natural active molecule existing in plants, has attracted many clinical researchers to conduct related clinical trials, attempting to promote the clinical transformation process of PD. These clinical trials involve irritable bowel syndrome, pelvic pain, liver diseases, etc. In a multicenter trial involving three Italian pediatric gastroenterology centers, researchers selected children aged 10 to 17 as participants and used a double-blind, placebo-controlled, parallelod-group design approach to study and evaluate the efficacy and safety of co-micropowder palmitoyl glycolamide (PEA)/PD in the treatment of abdominal pain symptoms in children with irritable bowel syndrome. Through the analysis and comparison of the intensity and frequency of abdominal pain, stool characteristics, etc. of the participants, it was confirmed that the symptom relief rate of children receiving PEA/PD treatment was significantly improved [140]. Similar to this result, in the clinical trial conducted by Cremon et al. [141] with the European population as the research center, it was also confirmed that PEA/PD treatment could effectively alleviate the severity of abdominal pain in patients with irritable bowel syndrome. Also, a clinical trial study of PEA/PD have proved that PEA/PD is an effective adjunct therapy for primary dysmenorrhea in adolescent girls. In this study, 110 patients with primary dysmenorrhea took 400 mg ± 40 mg of PEA/PD orally daily. Compared with 110 patients who took a placebo orally, the safety and efficacy of PEA/PD were confirmed [142]. Monte et al. [143] evaluated the impact of PD on chronic pelvic pain related to endometriosis in 30 symptomatic women who were eager to conceive. The patient was given 400 mg ± 40 mg PEA/PD twice a day for 80 days. The results showed that these treatments had a protective effect and effectively improved the pain symptoms, psychological status and quality of life. It is worth noting that in the above-mentioned clinical trials on PEA/PD, no adverse events were reported. These findings preliminarily suggest that PEA/PD has a good safety profile. For 20 alcoholics hospitalized for rehabilitation treatment, researchers compared the therapeutic effects of two nutritional strategies, namely vitamin C and vitamin C combined with PD. They found that PD reduced the aspartate aminotransferase (AST), lanine aminotransferase (ALT) levels and lipid peroxidation levels of the patients, which confirmed the potential of PD preparations as liver-protecting tablets [144]. The research by Fuggetta et al. [145] indicates that for patients with mutant non-small cell lung cancer taking afatinib, local application of 1.5% PD cream twice daily can effectively reduce the incidence of skin toxicity.

The various clinical trials mentioned above have confirmed that PD is a good choice for the treatment of chronic pelvic pain, irritable bowel syndrome and liver diseases, and is a reasonable subject for conducting clinical trials. This also brings benefits to the commercial development of PD. This can be concluded from the commercial development of resveratrol. Due to the relatively sufficient clinical research on resveratrol, many related products have already been launched on the market. The “Paradise Herbal Resveratrol” or “Longevity Resveratrol” in the United States are both medicines containing resveratrol. In Japan, resveratrol is extracted from Polygonum cuspidatum as a functional food additive. In China, products such as “Tianshi Huoluokang Capsules” from Tianjin Tienshi Group, “Zijin Capsules” from Sichuan Kanggaoxin Pharmaceutical Co., Ltd., and “Nabei Yisheng Capsules” from Xi 'an Nabei Biotechnology Co., Ltd. are all health care products containing resveratrol. With the advancement of clinical trials of PD, the application of PD is becoming increasingly widespread. Shenzhen Haiwang Bioengineering Co., Ltd. extracted and purified PD from Polygonum cuspidatum and independently developed Polygonum cuspidatum injection for the treatment of cardiovascular diseases. In recent years, due to the increasing demand for Polygonum cuspidatum, wild Polygonum cuspidatum and cultivated Polygonum cuspidatum have been allowed to be used in combination. Moreover, the Chinese Pharmacopoeia stipulates that the content of PD in dried medicinal products must not be less than 0.15%, which indicates that the medicinal value of PD is receiving more and more attention.

But more opportunities and challenges also follow. First of all, at present, both PD and drugs developed based on PD are in the preclinical trial stage. Moreover, the development of natural products involves a series of complex procedures such as extraction, refining and transformation, which increases the difficulty for PD to meet the clinical transformation standards. Secondly, the pathophysiological process of SCI is complex, and a single treatment cannot achieve the expected standard. In clinical practice, patients with SCI are confronted with a series of serious risks such as ischemia, hypoxia and paraplegia. Therefore, it is relatively difficult to conduct clinical research on the treatment of PD in SCI. These circumstances imply that there is still a long way to go to extract molecular PD from the natural Chinese medicine Polygonum cuspidatum for the treatment of SCI and it is not easy to truly commercialize PD. Both drug delivery issues and drug trial problems will become stumbling blocks in the clinical transformation process of PD. But we always believe that as more and more interested experts pay attention to these and more and more high-quality research is continuously implemented, PD is bound to make outstanding contributions to improving human health.

## 6 Conclusion

PD is a natural stilbene compound derived from knotweed. Experimental studies consistently show that PD shows significant potential in the treatment of SCI through its multi-target mechanisms of antioxidant stress, anti-inflammatory, and neuroprotective effects. Compared to traditional treatments such as methylprednisolone, PD offers a safer, more versatile treatment option with minimal toxicity and a wide range of pharmacological activities. However, several challenges remain to be addressed before its clinical application. Novel drug delivery systems, synergies between treatment strategies, and rigorous clinical trials all need to be explored. With continued research and technological advances, PD may pave the way for a more effective and comprehensive approach to SCI management.

## CRediT authorship contribution statement

**Zhishuo Wang:** Writing – review & editing, Writing – original draft. **Jiaming Zhang:** Writing – original draft. **Longyu Li:** Writing – review & editing. **Yuhao Zhang:** Writing – review & editing. **Haoyu Shen:** Writing – review & editing. **Chunfeng Shang:** Writing – review & editing. **Zikuan Leng:** Writing – review & editing. **Guowei Shang:** Writing – review & editing. **Hongwei Kou:** Writing – review & editing. **Keya Mao:** Writing – review & editing. **Hao Han:** Supervision. **Songfeng Chen:** Supervision, Funding acquisition. **Hongjian Liu:** Supervision, Project administration, Funding acquisition.

## Declaration of competing interest

The authors declare that they have no known competing financial interests or personal relationships that could have appeared to influence the work reported in this paper.
